# A holocene *n*-alkane stable isotope record from Wonderwerk Cave, South Africa and its implications for the later stone age record

**DOI:** 10.1038/s41598-025-99054-0

**Published:** 2025-04-26

**Authors:** Michaela Ecker, Sara Rhodes, Nils Andersen, Liora Kolska Horwitz, Michael Chazan, Cheryl A. Makarewicz

**Affiliations:** 1 Institute of Prehistoric and Protohistoric Archaeology, Kiel University, 24118 Kiel, Germany; 2 Interdisciplinary Center for Archaeology and Evolution of Human Behaviour, Universidade do Algarve, 8005-139 Faro, Portugal; 3 Leibniz Laboratory for Radiometric Dating and Stable Isotope Research, Kiel University, 24118 Kiel, Germany; 4 National Natural History Collections, Hebrew University, 91904 Jerusalem, Israel; 5 Department of Anthropology, University of Toronto, Toronto, ON M5S 2S2 Canada; 6 Evolutionary Studies Institute, University of the Witwatersrand, Johannesburg, 2000 South Africa

**Keywords:** Holocene, *n*-alkanes, Carbon isotopes, Hydrogen isotopes, Later Stone Age, Palaeoclimate, Biogeochemistry

## Abstract

Sediment biomarkers are important archives of regional, and global climate signatures, particularly in regions which lack continuous terrestrial archives such as the semi-arid deserts of Africa. We measured carbon and hydrogen stable isotopes from plant wax *n*-alkanes recovered from the Holocene Later Stone Age (LSA) sedimentary sequence at Wonderwerk Cave (South Africa), that comprises several technocomplexes (Kuruman/Oakhurst, Wilton, Ceramic Wilton and Historic). The plant wax *n*-alkane results were integrated with published palaeoenvironment reconstructions from the cave based on faunal, botanical, geological and sedimentological proxies which provides a robust comparative framework. The findings match well with other proxy records from the same strata and indicate a semi-arid to semi-humid early Holocene, with a mix of woody C_3_ plants and C_4_ grasses. In contrast, the mid-late Holocene environment was increasingly arid, open and dominated by C_4_ grasses. A distinct humid period at 5300–6200 cal. BP is evident, associated with a high density of archaeological material and a change in cultural expression in the Wonderwerk record. This study provides a step forward in using stable isotopes from biomarkers to create terrestrial environmental records in semi-arid regions.

**Supplementary Information:**

The online version contains supplementary material available at 10.1038/s41598-025-99054-0.

## Introduction

Correlations between local environmental change and cultural markers are usually investigated through the integration of environmental proxy studies and archaeological analysis at a local or regional scale. Firmly correlating past climatic shifts and cultural expression is key to expanding our understanding of human cultural development [e.g.^1,2^]. However, to disentangle the expression of macro-climatic versus local environmental changes, as would have been experienced by past human populations, requires a multi-proxy approach, since each proxy record reflects only specific parameters of past conditions. Sediment biomarkers, such as plant wax *n*-alkanes and their isotopic compositions, are an important source of multi-scalar climate signals, particularly in areas where long-term terrestrial archives are lacking [e.g.^3,4^]. With their ability to persist across geological time periods^5,6^, plant wax *n*-alkanes have enormous potential to provide environmental information on a molecular level since large parts of Southern Africa have minimal organic macroscopic (e.g. faunal remains) and microscopic (e.g. pollen) preservation. This hold true for many Later Stone Age (LSA) archaeological sites spanning the last 46,000 years in the interior of South Africa^7^. Three studies on southern African sites have measured carbon and hydrogen stable isotopes from plant wax *n*-alkanes derived directly from specific archaeological layers [e.g.^8–10^], but none have included sites in the arid interior of the country.

**Fig. 1:**
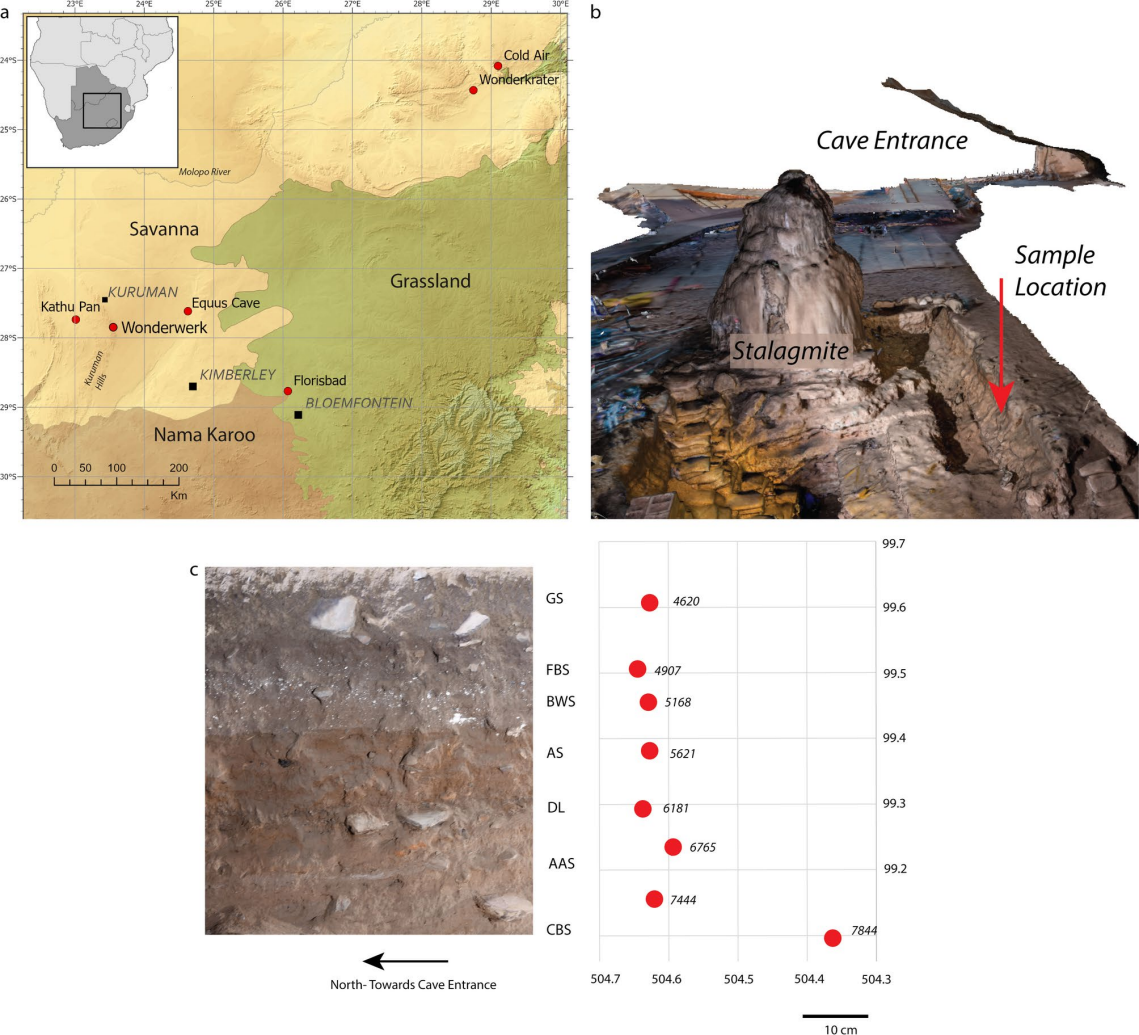
Location of sites and sample context. (**a**) Location of Wonderwerk Cave and surrounding sites within the vegetation biomes of South Africa as well as locations of comparable environmental records; (**b**) Scan of the Wonderwerk Cave entrance with view of the profile of operation 5, Excavation 1 (courtesy the Zamani project, University of Cape Town); (**c**) Picture of profile during excavation and location of the biomarker samples in the corresponding square L510d and L509a (sample from CBS). Samples were taken from the buckets during excavation.

The LSA in the arid interior of South Africa is characterised by short term occupations of both caves/rockshelters and open-air sites^11–15^, that imply either shifting human demography or reflect specific adaptational strategies to changing environmental conditions. Lithic technologies reflect a high degree of standardization across broad geographic ranges suggesting extensive social networks through time^13,15–17^. Long distance transport of lithic raw materials^15^ and ostrich eggshell beads^18^ further attest to the interconnected nature of regional LSA groups. Key to our understanding of the full LSA regional variability in this region is a precisely dated environmental record. Wonderwerk Cave, with a near-continuous LSA record of occupation from c. 12 ka to 500 BP, is a key reference site both for the archaeology and palaeoenvironment of the region (Fig. 1a, Supplementary text)^13,19^. Past work at the cave has shown a correlation between periods of wetter than present conditions and increased site use as reflected in artifact density measures^13^.

In the current study, we tested the application of plant wax *n*-alkane stable isotopes on Holocene sediment samples, taken during excavations at Wonderwerk Cave. We present the results of the Holocene layers from Excavation 1, Operation 5 at Wonderwerk Cave, which holds a rich, well dated Later Stone Age (LSA) archaeological record (Supplementary text) that encompasses Wilton, Ceramic LSA and historic cultural layers (Table 1, Table S1)^13^. The Holocene layers of Wonderwerk Cave Excavation 1 were first excavated in the 1980s^11^ and subsequently in 2018–19^13^. Strata from both excavations have been subjected to palaeoeonvironmental reconstruction using a broad spectrum of materials and methods; analysis of pollen and stable isotopes of speleothems^20^, charcoal identification^21,22^, pollen analysis from sediment^23^, taxonomy of micro and macro-mammal assemblages^24,25^, investigation of ostrich eggshell and herbivore stable isotopes^26,27^, and examination of tortoise remains^28^.

**Table 1:** Context information and biomarker parameters for the Wonderwerk Cave sediment samples. Correlation of the archaeological layers in the new excavation^13^ with the old excavation strata^11^ is given in table S1. The date ranges are based on the *Date*-function in the new age model (Dataset S2). For calculation of indices see supplementary information.

Sample number	Archaeological layers	Technocomplexes	Dates (cal. BP)	Sum (ng/µl)	Norm31	Norm33	ACL_27 − 33_	ACL_25 − 35_	CPI_27− 33_	OEP_25 − 35_
4723	Cave entrance	–	–	744.13	0.75	0.58	30.8	30.7	6.7	6.7
4620.5	GS	Historic, with glass	< 100 yrs. ago	3011.11	0.73	0.53	30.8	30.7	9.1	9.0
4907.5	FBS	Wilton, with pottery	680–2500	525.85	0.73	0.60	30.9	30.9	10.4	10.3
5168.5	BWS	Wilton, with pottery	2600–4540	416.42	0.73	0.59	30.9	30.9	10.5	10.3
5621.5	AS	Wilton	4580–5140	188.92	0.71	0.60	30.9	40.0	10.2	10.0
6181.5	DL	Wilton	5330–6280	457.04	0.69	0.50	30.4	30.2	13.2	12.8
6765.5	AAS	Wilton	6010–6810	837.36	0.74	0.55	30.6	30.6	12.3	12.2
7444.5	AAS	Wilton	6010–6810	426.80	0.75	0.57	30.7	30.7	11.0	10.8
7844.5	CBS	Wilton	6630–8440	189.20	0.78	0.63	30.9	30.9	7.6	7.4

Wonderwerk Cave is currently situated with the Savanna biome, more specifically the Kuruman Mountain Bushveld (SVK10), in close proximity to both the Nama Karoo and the Grassland biome (Fig. 1a)^29^. The local vegetation comprises of grasses such as *Themeda triandra*, *Cymbopogon plurinoides* as well as *Aristida* spp and some small trees and shrubs such as *Rhus* spp., *Tarchonanthus camphoratus*, *Acacia* spp., *Olea europaea* ssp. *africana*, *Grewia flava* and *Boscia albitrunca*^30^. The vast majority of grasses in the Savanna biome region of South Africa use the C_4_ photosynthetic pathway, while trees and shrubs use the C_3_ photosynthetic pathway^29^. Therefore, it is possible to trace changes in the proportions of grass and tree/shrub vegetation through the application of stable carbon isotope measurements (Supplementary Text)^31^. Hydrogen stable isotopes document water source, rainfall intensity, altitude and temperature amongst others^32^. Additionally, hydrogen stable isotopes are associated with differences between plant ecological lifeforms^33^. In the summer rainfall zone of South Africa specifically, where Wonderwerk Cave is located, the amount of rainfall^34^ and/or evapotranspiration^35^ have been proposed as the main influences on hydrogen stable isotope values. Today, the Wonderwerk Cave surroundings are dry to semi-arid with an average annual rainfall of 420 mm. Over two thirds of the rainfall occurs during the summer months^36^. Frost is common on some winter nights most years.

The Holocene sequence comprised a total of eight lithostratigraphic layers spanning from historic times to the early Holocene (Table 1, for details see^13^). This study was conducted on nine sediment samples collected during the 2019 excavation season at the cave (Table 1, for details see methods section) that span the Holocene sequence encompassing the Wilton (8440 − 4580 ca. BP), Ceramic LSA (4540 − 680 cal. BP) and historic cultural layers (< 100 years) in the Operation 5 profile (Table 1, Table S1). The ages of these layers were newly modelled here by combining the radiocarbon dates in^37] and [13^.

## Results

The new modelled age ranges for the radiocarbon dates concur with previously published age ranges (Table 1)^37^. Three additional dates from^13^ are in good agreement with the model and do not show any posterior outlier probability, while sample 6319 (layer DL) exhibits 7% posterior outlier probability in the outlier model. Despite this, the modelled age range of layer DL has not changed significantly within this time frame. Overall, the new model’s results have tightened the age ranges of all layers and better constrained layers BWS and FBS, highlighting that they are separate units.

The relative abundance of different biomarker chain lengths can reflect shifts in biome changes over time. The plant wax biomarker distributions show a clear odd-over-even ratio (Figure S1, Dataset S1). Higher chain lengths dominate indicating a terrestrial plant wax source with average chain length (ACL_27 − 33_) ranging from 30.4 to 30.9 (average 30.8 ± 0.18, *n* = 9) (Table 2), showing very little variation. If considering more homologues, ACL_25 − 35_ is almost identical at a range from 30.3 to 31.0 (Table 2). All samples display C_31_ as the dominant *n*-alkane, while the C_29_ and C_33_ homologues are the second most abundant, depending on the sample. This is consistent with reports of ACL from modern South African plants^31,38^. The carbon preference index (CPI) of the C_25 − C35_ *n*-alkanes ranges between 6.7 and 13.2 (10.1 ± 2.1, *n* = 9) (Table 2).

**Table 2:** Stable isotope results for the Wonderwerk cave sediment samples. All results are given as the average value of all measurements per sample in per mill (‰), with the δ^13^C values given against VPBD, δD values are given against VSMOW. The standard error of the mean (±SEM) is provided and the number of measurement are given in brackets. Not all samples had a high enough *n*C_27_ *n*-alkane concentrations to measure stable isotopes.

Sample number	Archaeological layers	δ^13^C *n*C_27_	δ^13^C *n*C_29_	δ^13^C *n*C_31_	δ^13^C *n*C_33_	δD *n*C_27_	δD *n*C_29_	δD *n*C_31_	δD *n*C_33_
4723	Modern cave entrance	− 25.58 ± 0.08 (3)	− 28.63 ± 0.01 (3)	− 28.89 ± 0.01 (3)	− 27.79 ± 0.02 (3)	− 153.8 ± 0.5 (4)	− 141.5 ± 1.7(8)	− 144.8 ± 1.2(8)	− 143.8 ± 0.6(8)
4620.5	GS	− 24.26 ± 0.04 (3)	− 28.50 ± 0.02 (3)	− 29.09 ± 0.01 (3)	− 27.65 ± 0.01 (3)	− 150.0 ± 0.8 (4)	− 147.0 ± 0.1(4)	− 147.7 ± 0.6(4)	− 145.4 ± 0.4(4)
4907.5	FBS	− 24.05 ± 0.03 (3)	− 27.51 ± 0.04 (3)	− 27.16 ± 0.04 (3)	− 23.61 ± 0.04 (3)	− 160.8 ± 0.7(4)	− 146.6 ± 0.6(4)	− 142.0 ± 0.6(4)	n/a
5168.5	BWS	n/a	− 27.47 ± 0.05 (3)	− 27.42 ± 0.02 (3)	− 23.91 ± 0.05 (3)	− 168.1 ± 1.0(2)	− 151.2 ± 2.5(3)	− 150.4 ± 1.3(3)	− 152.0 ± 0.0(3)
5621.5	AS	n/a	− 26.86 ± 0.33 (3)	− 26.74 ± 0.04 (3)	− 23.89 ± 0.09 (3)	− 142.4 ± 1.2(4)	− 141.6 ± 0.4(4)	− 150.3 ± 0.3(4)	− 149.8 ± 1.4(4)
6181.5	DL	− 23.30 ± 0.04 (3)	− 25.53 ± 0.05 (3)	− 25.83 ± 0.02 (3)	− 23.61 ± 0.05 (3)	n/a	− 153.0 ± 0.1(4)	− 152.0 ± 0.2(4)	− 142.2 ± 0.5(4)
6765.5	AAS	− 23.55 ± 0.02 (3)	− 25.94 ± 0.02 (3)	− 26.50 ± 0.01 (3)	− 24.74 ± 0.03 (3)	− 185.9 ± 1.0(4)	− 155.7 ± 2.2(4)	− 145.6 ± 0.6(4)	− 142.8 ± 0.1(4)
7444.5	AAS	− 23.72 ± 0.06 (3)	− 26.22 ± 0.01 (3)	− 26.70 ± 0.02 (3)	− 25.21 ± 0.05 (3)	− 183.5 ± 0.4(4)	− 158.7 ± 1.3(4)	− 144.5 ± 0.2(4)	− 144.2 ± 0.2(4)
7844.5	CBS	− 23.93 ± 0.08 (3)	− 26.34 ± 0.05 (3)	− 26.54 ± 0.04 (3)	− 25.44 ± 0.15 (3)	− 143.4 ± 1.0(4)	− 149.2 ± 0.5(4)	− 147.7 ± 0.3(4)	− 145.5 ± 0.9(4)

**Fig. 2:**
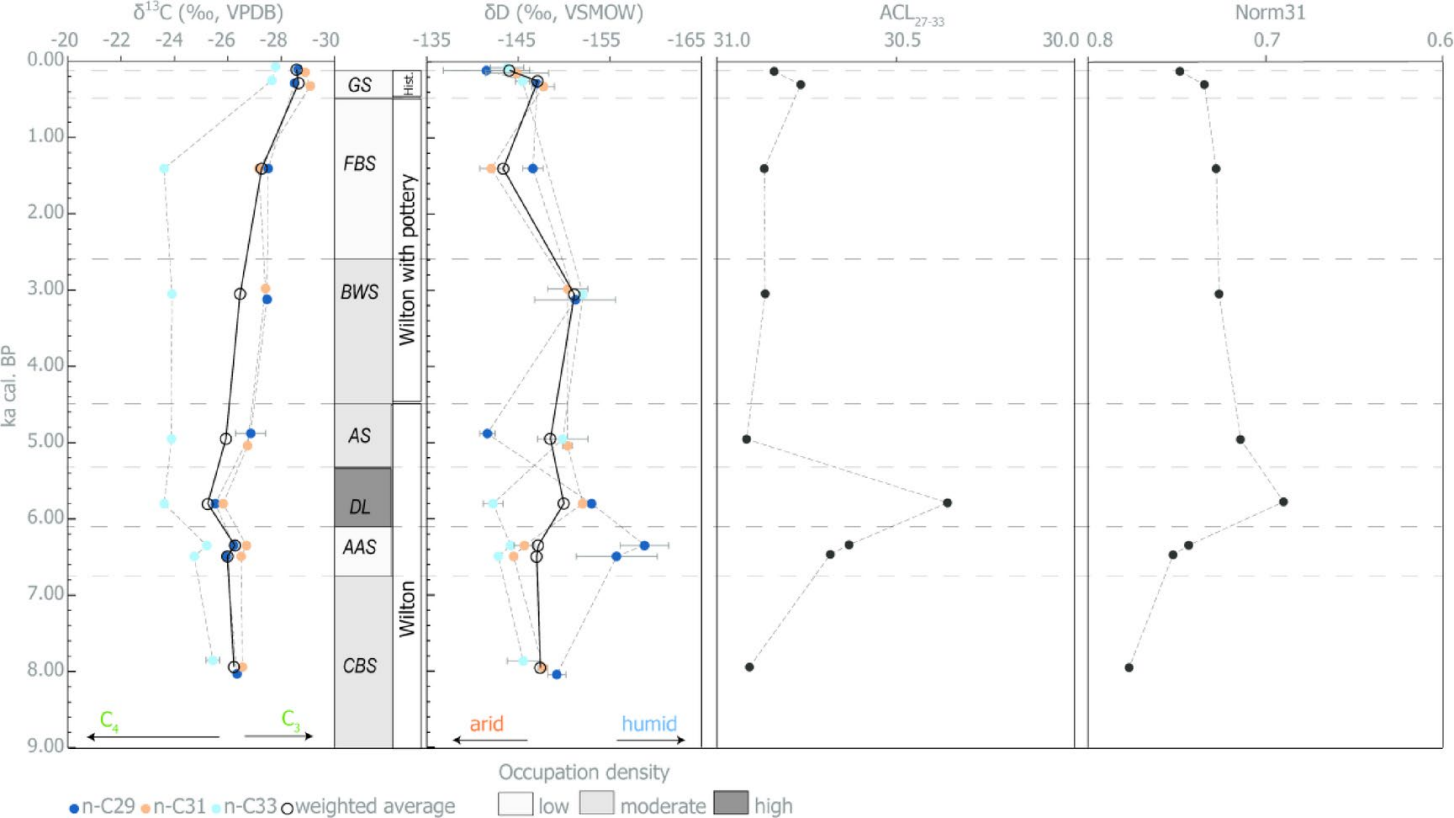
Distribution of biomarkers from Wonderwerk Cave sediment and their carbon (δ^13^C) and hydrogen (δ^2^D) composition. From left to right: Timeline in ka cal. BP; C_29_, C_31_ and C_33_ δ^13^C values with error bars (± 1SD) and their weighted average; name of stratigraphic layer with estimated occupation density based on quantity of cultural material; archaeological complex; C_29_, C_31_ and C_33_ δD values with error bars (± 1SD) and their weighted average; average chain length (ACL) of the C_27_-C_33_ *n*-alkanes; Norm31 index.

The carbon and hydrogen stable isotope results for C_27,_ C_29,_ C_31_ and C_33_ are given in Table 2, and the full result can be found in Dataset S1. A sediment sample from a fenced off area in the modern cave entrance gave δ^13^C values in the C_3_ plant range of − 28.6‰ (C_29_) and − 28.9‰ (C_31_) and δD values of − 141.5‰ (C_29_) and − 144.8‰ (C_31_). We did not correct the modern cave entrance samples or sample GS (< 100 yrs. ago) for the Suess effect as we do not know their exact time depth. Any correction would be based on a guessed value, and their corrected value (up to a maximum of 2‰) would still fall within the range of the archaeological samples. However, we have taken this into account in our statistical analysis (Table S2). The archaeological samples δ^13^C results for the C_29_, C_31_ and C_33_ exhibit comparable isotopic signatures throughout the sequence (Fig. 2) with the C_33_ *n*-alkanes displaying the highest δ^13^C values throughout. The δ^13^C values range from − 25.5‰ to − 28.5‰ in C_29_ and from − 25.8‰ to − 29.1‰ in C_31_. In contrast, no chain length consistently shows the highest δD values. For example, δD ratios for C_29_ anti-phased from the trend of the δD values for C_31_ and C_33_. The δD values range from − 141.6‰ to − 158.7‰ in C_29_, and from − 142‰ to − 150.4‰ in C_31_ for the archaeological samples, which overlaps with the sample from the modern cave entrance.

## Discussion

The modern comparative sample from the cave entrance (Sample 4723) reflects the vegetation of the Ghaap plateau today, which has been heavily altered by anthropogenic activities, including overgrazing and the planting of non-native species. Overgrazing and currently high CO_2_ levels are advantageous for C_3_ plants, which could explain the signal in the isotopic C_3_ plant range for the modern and historic sample (sheep were also held in the cave during historic times). The opening of the cave faces north toward the Ghaap plateau which is most likely the main source of the plant wax recovered. This is confirmed by the sediments from inside the cave from Operation 5, which are primarily comprised of quartz sands of aeolian origin matching Kalahari derived sands that accumulated across the landscape outside the cave^13,39^. Another potential source of plant wax is via water percolation through the banded ironstone and dolerite formation into the cave. Notably, there is a large stalagmite and various flowstone deposits in close proximity to Operation 5^20,40^, however, we did not sample any sediments within the flowstone deposits. Moreover^40^, have shown that the large speleothem in the cave entrance was not growing between 13 ka and 3.5 ka, suggesting very little infiltration of water into the cave over the duration of the period we are researching. It cannot be ruled out that humans and/or animals introduced selected plant material into the cave in the past, (e.g. for fuel, bedding, food and crafts), which served as a source, at least in part, of the plant wax^8^. However, irrespective of the vector of the botanical remains, we consider the resulting data to reflect local environmental conditions in the past. This is borne out by the fact that our results match the other environmental proxies from the same layers of Wonderwerk Cave, including those which track a range of parameters in addition to vegetation cover. This implies that the plant wax data are reliable in reflecting the local to regional Holocene palaeoenvironment.

Burning is a potential source of diagenetic alteration of plant waxes, however, the CPI values indicate that no significant plant wax degradation occurred^41,42^ despite the variable but clear presence of charcoal in the Holocene layers we sampled^22^ and of faunal bone specimens (5–15% across all layers) bearing traces of burning. Additionally, the odd-over-even predominance (OEP_25 − 35_) ranges from 6.4 to 12.8 for the archaeological samples (Table 1), suggesting little degradation through burning or other processes of organic matter degradation^43^.

The relative abundance (Figure S1) of *n*-alkane biomarkers can be used to determine the proportional sedimentary input of woody C_3_ plants and C_4_ grasses. All samples show the dominance of C_31_, followed by C_33_ or C_29_. These results, as well as ACL_**27 − 33**_ and CPI_25 − 35_ indices, most closely match the signature of Nama Karoo samples obtained by^38^, indicating a source vegetation at Wonderwerk that is similar to today’s Nama Karoo biome. The exception to this signal is the sample from layer DL (Table 1; Fig. 2), where C_29_ and C_33_ chain lengths are almost equal, indicating a higher proportion of C_3_ plants than in the other layers. However, Herrmann et al.’s^38^ study included only a small number of Savanna biome samples compared to the other South African biomes, and more samples from the wider Savanna biome region are necessary to refine these values. Additionally, ecological lifeform, aridity, and temperature can influence dominant chain length, while the δ^13^C results are a direct proxy for photosynthesis.

**Fig. 3:**
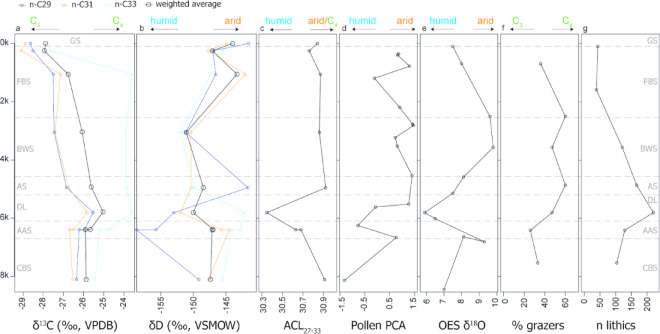
Proxy records from the Holocene strata at Excavation 1, Wonderwerk Cave. Timeline in ka cal. BP. (**a**) C_29_, C_31_ and C_33_ δ^13^C values and their weighted average; (**b**) C_29_, C_31_ and C_33_ δD values and their weighted average; (**c**) average chain length (ACL) of the C_27_-C_33_ *n*-alkanes; (**d**) Pollen PCA based on^23^; (**e**) ostrich eggshell δ^18^O values based on^26^; (**f**) percentage of grazers in the faunal assemblage based on enamel δ^13^C values from^27^; number of lithics excavated in Operation 5 from^13^.

The δ^13^C *n*C_31_ results between − 25.83‰ and − 29.09‰ generally indicate a mix of C_3_ and C_4_ plants in the environment. The δ^13^C values of *n*C_29_, *n*C_31_ and *n*C_33_ overall, as well as their weighted average, show the same trends, especially in the early Holocene (layers CBS, AAS and DL), with an increase in C_4_ plant input from CBS to AAS and further to layer DL. In the mid-Holocene layers AS and BWS, *n*C_33_ does not decrease to the same extend as the other chains and the weighted average. As *n*C_33_ is particularly representative of C_4_ grasses^42^, this could indicate a strong C_4_ grass presence from the mid-Holocene onwards as has been proposed by other proxies from Wonderwerk Cave (Fig. 3).

The highest variability as well as the largest error bars are seen in δD *n*C_29_ (Fig. 2). It shows opposite trends in moisture availability in the early to mid-Holocene (layers CBS, AAS, DL) but better aligns with the *n*C_31_ and *n*C_33_ trends seen in later levels. *n*C_29_ indicates increased humidity in layers AAS and DL while *n*C_33_, indicative of C_4_ grasses, indicates increased aridity. The anti-phased trends in the chain length δD isotope values, might indicate changes in either the relative proportions of C_3_ and C_4_ plants (in the environment or in plants humans brought into the cave), the water stress experienced by each plant type, or a change in plant type, in amount of rainfall or rainfall regime^32^. If the weighted average is taken as measure for overall changes in aridity and our results demonstrate more humidity in mid-Holocene layers DL, AS and BWS, with DL being the most humid. The *n*C_31_ values match closely the trends in the weighted average of *n*C_29_, *n*C_31_ and *n*C_33_ (Figs. 2 and 3), which shows a wetter mid-Holocene (layers DL-BWS) compared to the layers in the early and late Holocene. Additionally, the indices Norm31 and Norm33, which have been tentatively linked to environmental parameters such as water stress^31,44^, are lowest in layer DL (Table 1; Fig. 2). Higher ACL have been interpreted as an indicator of more grasses and/or drier conditions in the arid areas of southern Africa^45^, and following this interpretation, layer DL stands out as representing a more humid phase. All these results indicate less water stress and potentially also more C_4_ plants in layer DL, which is dated to ~ 5330–6280 cal. BP, and thereafter.

The biomarker results are in good agreement with other environmental proxies regarding general trends in vegetation and climatic conditions in the Holocene layers of Excavation 1 at Wonderwerk Cave (Fig. 3)^22–24,26–28^. The results show an early Holocene (layers CBS and AAS) with a mix of woody C_3_ plants and C_4_ grasses in contrast to an increasingly C_4_ grass dominated later Holocene (Fig. 3). The faunal assemblage reflects this by the presence of Tragelaphini and a reduced number of Equids in layers CBS and AAS compared to the younger layers (Fig. 3), the percentage of predominantly C_4_ grazers in these layer is the lowest in the whole sequence^27^. The last appearance of the extinct *Antidorcas bondi* and *Megalotragus priscus* in layer CBS, hints at a different palaeoecological structure, including remnants of the Pleistocene grazing succession^27^. The pollen data of the same layers^23^, provides further corroboration for the interpretation of a dry karroid vegetation in the early Holocene. A shift in pollen^23^ as well as tree-cover based on charcoal taxa analysis^22^, indicates a change to more humid grassy conditions in the mid-Holocene, starting in layer AAS and reaching its most extreme after c. 6200 cal. BP (layer DL), which has been taken as indication of a shift in rainfall regime^23^. This trend is also mirrored in tortoise body mass, which following changes in plant productivity, was lowest in the drier early Holocene layers, but increased significantly in the moister mid-Holocene layers^28^.

The δD C_29_ and C_31_ alkanes are an additional line of evidence showing more humidity in layers AAS and DL. Divergent trends in the chain length δD isotope values are especially strong between C_33_ (representing mainly C_4_ grasses) and C_29_ (representing mainly C_3_ plants). Modern δD Savanna biome sediment values range from − 119 ± 0‰ to -103 ± 3 for δD in C_29_ and − 141 ± 1‰ to -123 ± 1‰ for δD in C_31_^34^. The sample from the current Wonderwerk cave entrance is more negative (-141.5 ± 1.7‰ for C_29_ and − 144.8 ± 1.2‰ for C_31_), as is entire range of Wonderwerk δD values compared to the modern ones. Indeed, the Wonderwerk δD values fall within the Grassland biome range of^34^. However, the samples in that study were not taken in the Wonderwerk Cave region but further south. If compared to the range of the modern summer rainfall zone as a whole, the Wonderwerk Cave values all fall within these ranges. Another local line of evidence for past humid conditions comes from the ostrich eggshell δ^18^O record from Wonderwerk Excavation 1^26^. Further evidence can be found in the summer rainfall zone, where a mid-Holocene humid period is recorded from studies of the Cold Air Cave stalagmite^46^, Equus Cave pollen and ostrich eggshell stable isotopes^47,48^, Wonderkrater pollen record^49^, Kathu Pan 6 sediments^50^ and the Florisbad pollen record^51^. Overall, a rise of C_4_ grasses following a wet phase is conclusive as reflected in the species composition of micro and macro-fauna, pollen and the biomarker record at Wonderwerk Cave, with C_4_ grasses remaining more abundant in the subsequent mid-late Holocene (layers AS, BSW, FBS). Higher δD values, comparable to modern δD values^34^ across all chain lengths within the late Holocene layer FBS, indicate an arid phase in the last 2000 years which is in agreement with the pollen data. Alcelaphini and Equids, both grazing species dominate the faunal record in layers AS and FBS.

Small fluctuations in aridity are highly likely in the late Holocene, especially in layer BWS, but are less prominent and poorly time-constrained. Layer DL stands out as a turnover point for the expansion of the C_4_ grassland. In parallel, it is associated with the highest density of archaeological material in the Wonderwerk Cave record. We detect a technological shift in the increased frequency of segments relative to backed bladelets and the use chert for stone tool manufacture in layers DL and AS, as well as changes in worked ostrich eggshell abundance from layer DL onwards. Similar shifts in technology and raw material are characteristic of Wilton lithic assemblages from South Africa’s interior, dating to c. 8-2 ka cal. BP (Supplementary Text)^12–14^.

There are significant correlations between the *n*C_27_, *n*C_29_ and *n*C_31_ δ^13^C values and the amount of lithics per layer (indicating occupation density), as well as between *n*C_27_ δ^13^C values and the number of recovered ostrich eggshell (OES) fragments (Table 3). The correlation between increased C_4_ plants in the environment and higher site occupation intensity and/or artifact density (Table 3), might reflect changes in human mobility or an adaptation to a changing botanical and faunal ecosystem. If corrected for the potential Suess effect by 2‰ there are fewer significant correlations (Table S2), however, especially for OES the correlations persist. The higher density of lithic artefacts, fauna and culturally modified OES, during and after the humid period of ~ 5400–6300 cal. BP, coincides with the growing dominance of the C_4_ grassland. However, the data on site occupation is more nuanced if sedimentation rate is taken into account. Layer DL has the greatest density of lithics/sediment and suggesting clear evidence for an increase in human activity during this period. However there is reason for caution as the rate of sediment accumulation is also a factor in the density of artifacts. When we take this factor into consideration (Figure S2) it appears that the high rate of lithic artifact deposition found in DL might continue into the overlying AS, where there is an increase in the rate of deposition (as calculated based on radiocarbon ages), but these two layers are both clearly distinct from underlying and overlying deposits. Considering this, it stands that there was increased human activity in the mid-Holocene at Wonderwerk Cave compared to previous and later layers and at least part of this is linked to wetter and/or more open environments. Increased human activity could also mean increased input from collected plant resources into the cave, which could potentially bias the biomarker results when human-selected plants overprint the signal of the natural environment as a whole. However, as our results are consistent with trends in other proxies from the site, some of which are independent of human plant collection, we assume the *n*-alkane stable isotope values are not influenced by increased human activity in the cave.

**Table 3:**
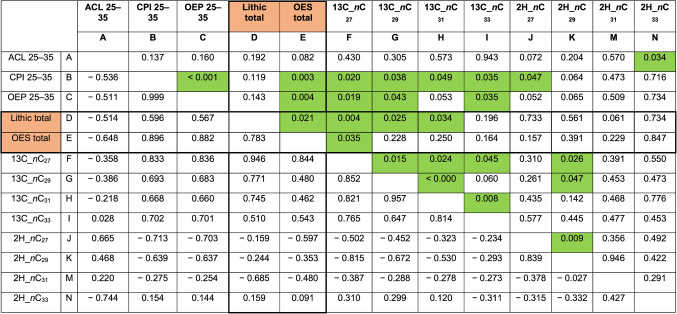
Linear R pearson test (bottom/left) and *p* values (top/right) for all samples separated by biomarker indices and isotope values, including numbers of lithic artefacts and ostrich eggshell (OES) pieces per layer.

Lithic and OES counts from^13^. Significant *p*-values (< 0.05) are in highlighted in green.

In the late Holocene, the initial occurrence of pottery at the site in layer FBS and BWS^11^ may reflect increased mobility of hunter-gatherer groups with new technologies at times of aridity. However, there does not seem to be a clear environmental change connected to the introduction of pottery at Wonderwerk Cave, since this was a widespread phenomenon throughout southern Africa, and most likely did not originate in our research region. Likewise, the introduction of sheep into southern Africa ca. 2000 BP, was a general phenomenon^52^. There is limited evidence for the presence of sheep at Wonderwerk Cave and this includes dung and associated spherulites identified in a micromorphology thin section^13^ and SEM identification of sheep hair^53^, both of which originate in layer FBS (dated to 680–2500 cal. BP), which hints at new subsistence practices.

Through the integration of environmental multi-proxy studies and high-resolution archaeological excavation we have demonstrated a close connection between local Holocene environment and cultural change at Wonderwerk Cave. Our study shows that sedimentary biomarkers can provide additional insights into local environmental conditions but demonstrate a complex picture that will require more focused research on the Northern Cape region to fully understand. Increased interest in this region and period will further highlight the complex lifeways of Later Stone Age hunter-gatherers and potentially improve our knowledge about, and cultural perception of, modern hunter-gatherers groups living in similar environments.

## Methods

### Sample collection

Eight sediment samples were taken during excavation in Excavation 1, Operation 5, located about 20 m in from the cave entrance. Additionally, one sample of the modern topmost layer at the cave entrance near the stalagmite (Fig. 1b–c) was taken for comparison. This location is within the fenced off archaeological research area not accessible to the public and was taken as a reference for the greater modern ecoregion. Excavation followed a high-resolution approach, excavating 0.25 × 0.25 m squares in 5 cm spits^13^. Samples were taken directly from the excavated sediment bucket during excavation and packaged in paper bags and aluminum foil. In the laboratory, samples were sieved in a 200 μm sieve and then homogenized by grinding in a mortar and pestle. 6 g of sediment from each sample was used for extraction of *n*-alkanes.

### Extraction of ***n***-alkanes

The sample preparation followed the protocol of the biomarker laboratory at Kiel University. The *n*-alkanes were extracted with an accelerated solvent extractor (ASE-200, Dionex) at 100 bar and 100˚C using a 9:1 (v = v) mixture of dichloromethane (DCM) and methanol. The *n*-alkanes were subsequently separated by silica gel column chromatography using activated silica gel and hexane. *n*-alkanes were further separated using silver nitrate (AgNO_3_) coated silica gel. Individual *n*-alkane homologues were identified with an Agilent 6890 N gas chromatograph equipped with a Restek XTI-5 capillary column (30 m x 320 μm x 0.25 μm) based on the comparison of their retention times with a standard containing the *n*-alkane homologues *n*-C_20_, *n*C-_24_, *n*-C_28_, *n*-C_32_, *n*-C_36_, *n*-C_38_ and *n*-C_40_ of known concentration. This standard was used to calculate a calibration factor for quantification of the samples and was additionally measured at the start and end of every batch of six samples. All the samples were measured in triplicates. *n*-alkanes were quantified using the FID peak areas multiplied with the calibration factor of the standard and the original sample weight. The following GC-FID temperature program was used: the oven was maintained for 1 min at an initial temperature of 50 °C, then the temperature was increased to 300 °C (10 min hold) before a further increase to 330 °C (10 min hold). Infromation on the calculation of indices (ACL, CPI, OEP, Norm31, Norm33) can be found in the supplementary information.

### Age model

We combined the previously modelled Wonderwerk radiocarbon dates from the Thackeray excavations^37^ with the four new radiocarbon dates from Operation 5 published in^13^. Dates from Thackeray’s Strata 4d and 5a were excluded, as these layers were not part of this study and have not been investigated in the new excavation. OxCal v4.4^54,55^ was used for Bayesian analysis to integrate the ^14^C data with the Operation 5 stratigraphic information, using the ShCal20 curve for the Southern Hemisphere^56^. We used a Sequence model, with Boundaries between the archaeological strata (prior information), and Phases within the Boundaries (Dataset S2). A general *Outlier_model* with prior outlier probability set to 5%^57^ was applied. The *Date* function was used to estimate the span of each strata, rather than using the modelled layer boundaries^37^. The modelled ^14^C date ranges are presented at 95.4% probability (approximately equivalent to 2σ uncertainty) and the ages are given in cal BP. See the Dataset S2 for the Model Code.

### Stable isotope measurements

Terrestrial-sourced *n*-alkane homologues of sufficient concentration (i.e. *n*-C_27_, *n*-C_29_, *n*-C_31_, and *n*-C_33_) were analysed using gas chromatography-isotope ratio mass spectrometry (GC-IRMS) for δD and δ^13^C at the Leibniz Laboratory for Radiometric Dating and Stable Isotope Research at Kiel University. Samples were measured on an Agilent 6890 gas chromatograph equipped with a Gerstel KAS 4 PTV injector and an Agilent DB-5 capillary column (30 m x 250 μm x 0.25 μm) coupled to a Thermo Scientific MAT 253 isotope ratio mass spectrometer (IRMS). Following GC temperature program was used for analyzing carbon isotopes: 50 °C for 5 min, with 20 °C/min to 325 °C, 325 °C for 18 min and those for hydrogen isotopes was: 50 °C for 5 min, with 40 °C/min to 240 °C, with 20 °C/min to 280 °C, with 10 °C/min to 325 °C, 325 °C for 20 min. Depending on the *n*-alkane concentration, between 0.3 and 1.0 µl and between 5.0 and 10.0 µl of each sample for δ^13^C and δD, respectively has been injected 2–4 times in order to achieve a statistically robust analytical error for each *n*-alkane homologue. To allow large volume injections (LVI), the injector was used in solvent vent mode. The H3 + factor during the measurement period of seven days was 10.33±0.11 ppm/nA (*n* = 7). The δD and δ^13^C values are reported relative to Vienna Standard Mean Ocean Water (‰ VSMOW) based on Arndt Schimmelmann’s A6 reference mixture from 2015 and Vienna Pee Dee Belemnite (‰ VPDB) scales using Arndt Schimmelmann’s A7 reference mixture from 2017, respectively. Statistical tests were run with PAST4.16c^58^.

## Electronic supplementary material

Below is the link to the electronic supplementary material.

Supplementary Material 1.

Supplementary Material 2.

Supplementary Material 3.

## Data Availability

All biomarker and stable isotope data related to the paper is available in Zenodo: 10.5281/zenodo.11219496. The OxCal code for the radiocarbon model is available in Zenodo. 10.5281/zenodo.11219496.
