# Supplementary material for: A holocene n-alkane stable isotope record from Wonderwerk Cave, South Africa and its implications for the later stone age record
Source: Sci Rep. 2025 Apr 26;15:14667. doi: 10.1038/s41598-025-99054-0 (PMC12033296; doi:10.1038/s41598-025-99054-0)
Supplement: Supplementary file 1 — Supplementary Material 1. [file 41598_2025_99054_MOESM1_ESM.pdf]

Supplementary Dataset 1 for

A Holocene n-alkane stable isotope record from Wonderwerk Cave, South Africa, and its implications for the Later Stone Age record

by M. Ecker, S. Rhodes, N. Andersen, L.K. Horwitz, M. Chazan, and C.A. Makarewicz

Code for radiocarbon model, used in OxCal 4.4.

```
Plot()
 Curve="ShCal20.14c";
{
 Outlier_Model("General",T(5),U(0,4),"t");
 Sequence()
 {
  Boundary("Start CBS");
  Phase("CBS")
  {
   R_Date("2545", 5970, 70)
   {
    Outlier(0.05);
   };
   R_Date("2798", 7430, 60)
   {
    Outlier(0.05);
   };
   R_Date("31897", 5063, 30)
   {
    Outlier(0.05);
   };
   Date("Date CBS");
  };
  Boundary("End CBS");
  Boundary("Start AAS");
  Phase("Phase AAS")
```

```
{
 R_Date("2544", 5180, 70)
 {
  Outlier(0.05);
 };
 R_Date("30640", 5627, 33)
 {
  Outlier(0.05);
 };
 R_Date("30641", 5771, 34)
 {
  Outlier(0.05);
 };
 R_Date("30642", 5915, 34)
 {
  Outlier(0.05);
 };
 Date("Date AAS");
};
Boundary("End AAS");
Boundary("Start DL");
Phase("DL")
{
 R_Date("2797", 4890, 70)
 {
  Outlier(0.05);
 };
 R_Date("30638", 5340, 33)
 {
  Outlier(0.05);
 };
```

```
 R_Date("30639", 4887, 33)
 {
  Outlier(0.05);
 };
 R_Date("6319", 5475, 34)
 {
  Outlier(0.05);
 };
 R_Date("7043.1", 4842, 55)
 {
  Outlier(0.05);
 };
 Date("Date DL");
};
Boundary("End DL");
Boundary("Start AS");
Phase("Phase AS")
{
 R_Date("6099", 4237, 28)
 {
  Outlier(0.05);
 };
 R_Date("6047", 4447, 30)
 {
  Outlier(0.05);
 };
 R_Date("2541", 4240, 60)
 {
  Outlier(0.05);
 };
 R_Date("30566", 4459, 30)
```

```
 {
  Outlier(0.05);
 };
 R_Date("30567", 4427, 29)
 {
  Outlier(0.05);
 };
 R_Date("30568", 4207, 30)
 {
  Outlier(0.05);
 };
 Date("Date AS");
};
Boundary("End AS");
Boundary("Start BWS");
Phase("Phase BWS")
{
 R_Date("2543", 2910, 60)
 {
  Outlier(0.05);
 };
 R_Date("2785", 3990, 60)
 {
  Outlier(0.05);
 };
 Date("Date BWS");
};
Boundary("End BWS");
Boundary("Start FBS");
Phase("FBS")
{
```

```
   R_Date("2779", 1210, 50)
   {
    Outlier(0.05);
   };
   R_Date("2542", 1890, 50)
   {
    Outlier(0.05);
   };
   R_Date("6873", 2120, 80)
   {
    Outlier(0.05);
   };
   Date("Date FBS");
  };
  Boundary("End FBS");
 };
};
```
