# Supplementary material for: A holocene n-alkane stable isotope record from Wonderwerk Cave, South Africa and its implications for the later stone age record
Source: Sci Rep. 2025 Apr 26;15:14667. doi: 10.1038/s41598-025-99054-0 (PMC12033296; doi:10.1038/s41598-025-99054-0)
Supplement: Supplementary file 3 — Supplementary Material 3. [file 41598_2025_99054_MOESM3_ESM.pdf]

Supplementary Materials for

A Holocene *n*-alkane stable isotope record from Wonderwerk Cave, South Africa, and its implications for the Later Stone Age record

Michaela Ecker[1]*, Sara Rhodes[2], Nils Andersen[3], Liora Kolska Horwitz[4], Michael Chazan[5,6], Cheryl A. Makarewicz[1]

1 Institute of Pre- and Protohistoric Archaeology, Christian-Albrechts University, 24118 Kiel, Germany

2 Interdisciplinary Center for Archaeology and Evolution of Human Behaviour, Universidade do Algarve, 8005-139 Faro, Portugal

3 Leibniz Laboratory for Radiometric Dating and Stable Isotope Research, Christian-Albrechts University, 24118 Kiel, Germany

4 National Natural History Collections, Hebrew University, Jerusalem 91904, Israel

5 Department of Anthropology, University of Toronto, Toronto, Ontario, M5S 2S2, Canada

6 Evolutionary Studies Institute, University of the Witwatersrand, Johannesburg 2000, South Africa

*Corresponding author Email: mecker@ufg.uni-kiel.de

This file includes:

Supplementary Discussion

Tables S1 to S2

Figure S1 to S2

SI references

Supplementary Discussion

Wonderwerk Cave and the Later Stone Age in the interior of South Africa

Wonderwerk Cave, situated in the Northern Cape Province of South Africa, is a large dolostone cave extending ca. 140 m into the Kuruman Hills. Located in the modern semi-arid savannah region, the cave contains an archaeological record ranging from the Early Stone Age through to the Holocene, including a phase of occupation by a local farmer and his livestock in the early 1900s [19]. Excavation of the Later Stone Age (LSA) deposits was initially undertaken in the 1940s by Malan and Cooke and Malan and Wells [59, 60]. Peter Beaumont reinitiated excavation of the LSA deposits in 1978 within Excavation 1, located directly behind the large stalagmite which marks the cave mouth. He was assisted by Anne and Francis Thackeray who extended his excavation and studied the lithic and faunal assemblages for their respective PhD theses [11, 25, 53, 61]. Beaumont's excavation continued from the 1980s onward with a focus on the Early Stone Age deposits. Unfortunately, many of Beaumont's LSA findings remain unanalyzed (for exceptions see [21-22, 27, 37, 62]). The Wonderwerk Cave LSA record became particularly well-known following the discovery of a number of incised stone slabs [61, 63] including the unfinished image of a large mammal relatively dated to 10,200±90 BP which, at the time, represented the earliest known sub-Saharan art mobilier [61]. The adornment of ~40m of the front cave walls with detailed rock art is also attributed to the LSA period, but at present no reliable method of dating this parietal art is available [64].

To address many of the contextual issues with the LSA assemblages from historic excavations at Wonderwerk Cave, new high-resolution excavations of a small (2m x 1m) exposed LSA profile was undertaken in 2018 [13]. By dividing this area into fourteen ¼ meter units and using modern excavation and provenience recording methods, precise 3D spatial distribution data was collected for all artifacts and ecofacts >1cm in maximum size. Sediment samples were taken from each lithostratific layer for leaf wax *n*-alkane analysis and future aDNA and lipid analysis along with continuous micromorphological block samples. Excavated sediment was water screened via a Flote-Tech floation machine (manufactured by R. Dausman [65]) which uses low-velocity water movement to separate botanical and microfaunal remains from the sedimentary matrix. From within this small excavation space a total of 3368 artifacts

and ecofacts were recovered and recorded of which 5 faunal remains exhibiting anthropogenic modifications (i.e. cut-marks) were selected for radiocarbon dating at the D-REAMS laboratory at the Weizmann Institute of Science, Israel.

The results of these renewed excavations demonstrate an excellent correlation with past studies of LSA assemblages from Wonderwerk Cave across all material types [13]. The newly produced radiocarbon chronology fits well within the previous Bayesian modelled chronology despite newly confirmed bioturbation of the LSA deposits by insect-sized organisms via micromorphological analysis. The authors correlated newly described sedimentary layers with the old excavation's stratigraphy (Table S1) and the new dates confirm a hypothesized short duration occupation of layer DL (previously 4aLH, for Living Horizon) of ca. 800 years. They also identify high amounts of banded ironstone formation as the cause of the dark discolouration within layer DL rather than previously hypothesized anthropogenic charcoal.

Like previous studies, the authors identify three technocomplexes within the Wonderwerk LSA sequence – the Kuruman/Oakhurst, Wilton, and a Historic unit with a glass component, each of which exhibit distinct shifts in raw material type and artifact density they propose correlates with shifts in site use and/or socioeconomic factors. Associated shifts in the density of anthropogenically derived faunal remains and both incised and non-incised ostrich eggshell fragments, also suggest increased occupation of the site during and after the deposition of layer DL. Ongoing studies of the newly recovered charcoal, pollen, and microfaunal assemblages will hopefully shed greater light on this variability in site use at Wonderwerk Cave throughout the LSA.

Such research is particularly important due to the paucity of well stratified LSA deposit from the region. Equus Cave, located in the nearby Ghaap escarpment, contains a 2.5-meter deep record of terminal Pleistocene and Holocene deposits marred by a periodically non-sequential radiocarbon chronology [66-67]. However, stable isotopic and amino acid racemization ostrich eggshell fragments do identify modern levels of precipitation around Equus cave as early as 6000 BP which agrees with the various proxy signals from Wonderwerk Cave indicating a mid-Holocene moist period [47]. Other LSA sites along the Ghaap escarpment contain deposits dating only to the late Holocene or with substantial chronological breaks spanning the time period of interest

[11]. By combining the multi-proxy paleoenvironmental signals of these LSA sites with a study of local tufa deposits [68], Humphreys and Thackeray [11] present a picture of late Pleistocene and Holocene climate along the Ghaap escarpment that includes cyclical shifts between arid/semi-arid conditions (~13500 – 11500 BP and 6500-4500 BP) and subhumid periods (9700-6500 BP and 4500 – 2400 BP) which, in some cases, directly contradict the signals identified in past studies of Wonderwerk Cave [20, 24-25]. Whether this discrepancy is due to differences and/or difficulties in correctly dating the Ghaap escarpment sites, differential response rates of various proxy records to climatic changes, or a localized response to the changing Holocene climate, remains to be investigated.

Redating efforts at Kathu Pan 6 confirm the presence of an LSA Wilton assemblage constrained via OSL dating to between 5700 and 2300 BP [69]. Recently published material records from this open-air site [14] report that the lithic assemblage is dominated by ironstone and chert, and the limited faunal remains preserved include multiple bovid size class 1 individuals alongside warthog and/or hippopotamus. The limited organic preservation at this sinkhole, which is located next to a perennial water source on the edge of the summer rainfall zone, make paleoenvironmental interpretations difficult. However, the 7-meter thick Florisbad spring deposits, located further south-east in the modern grassland biome, records a rich pollen record documenting episodes of increasing moisture availability at c. 6300 BP and between ca. 4500 and ca. 4000 BP, between more arid periods [51].

As this short summary of the paleoenvironmental information from the few LSA sites scattered across the Northern Cape makes clear, the Wonderwerk Cave records represents by far the best preserved paleoecological and archaeological archive in the region. Barring future discovery of long-duration, well preserved proxy records from this important prehistoric time period in the region, re-examination of the Wonderwerk and other local LSA records constitutes our best route to further refining our understanding of the relationship between terminal Pleistocene and Holocene environment and the lifeways of the hunter-gatherers which occupied these regions in the past.

Leaf wax n-alkanes as palaeoenvironmental indicators

Terrestrial plants produce a wax layer on their leaves as protection against water loss and UV radiation, amongst other factors. The leaf wax components pass into sediment from decaying plant matter as aerosols. A main component of leaf wax are long-chain *n*-alkyl compounds (*n*-alkanes, *n*-alcohols, *n*-alkonoic acids) [70]. These are environmentally persistent and can be recovered in sediments from geological time periods. *N*-alkanes are particular well suited to reconstruction of vegetation and hydrology as these biomarkers can be analyzed with compound specific stable isotope analysis. *n*-alkanes of terrestrial plants are characterized by the long-chain homologues C_{27}-C_{35}. The abundance of different chain lengths can tentatively be related to the photosynthethic pathway of the plant. Higher chains (C_{33}, C_{35}) are, for example, related to input from C_4 grass, whereas lower chain lengths are mostly related to input from C_3 plants like shrubs and trees [42].

Carbon isotope ($\delta^{13}C$) values reflect the abundance of C_3 and C_4 vegetation in the local environment. Due to their different photosynthetic pathways, C_4 plants typically exhibit elevated $\delta^{13}C$ values varying between -7 to -15‰ compared to those from C_3 plants, which vary between -20 to -35‰ for plant bulk samples [71]. Plant wax *n*-alkanes are further depleted from their bulk values by about -5 ‰ to -7 ‰ for C_3 vegetation and -8 ‰ to -10 ‰ for C_4 types. Therefore, depending on the plant ecological lifeform, water availability, canopy structure, and other factors, C_3 terrestrial plant *n*-alkane values can range from -25 ‰ to -42 ‰, while C_4 plants range from about -14 ‰ to -26 ‰ [72-75].

Hydrogen isotope (δD) values obtained from leaf wax *n*-alkanes record local meteoric water uptake and therefore reflect palaeohydrology [76]. Evapotranspiration leads to isotope enrichment in leafwater, which means that aridity is reflected in higher δD values in sediment samples. It has been shown that in the summer rainfall zone of South Africa, there is a correlation of δDwax and the amount of mean annual precipitation [34]. Further influences on precipitation and therefore ultimately δD values are temperature, amount of rainfall, rainfall character and transport from the precipitation source [32, 76-78].

Biomarker Indices

For molecular characterization of the gas chromatograph measurements several indices were calculated and their significance is discussed in the main paper.

Potential alteration of *n*-alkanes can be investigated by the carbon preference index (CPI) which has been calculated using the following formula:

$CPI_{25\text{-}35} = [(C_{25}+C_{27}+C_{29}+C_{31}+C_{33}) + (C_{27}+C_{29}+C_{31}+C_{33}+C_{35})] / [2x(C_{26}+C_{28}+C_{30}+C_{32}+C_{34})]$

As such, the average chain length (ACL) of *n*-alkanes might be used as a paleoclimatic indicator. Particularly for our study region, higher $ACL_{27\text{-}33}$ have been interpreted as an indicator of more grasses and/or drier conditions [45]. ACL was calculated using the following formula:

$ACL_{27\text{-}33} = (27 \times C_{27} + 29 \times C_{29} + 31 \times C_{31} + 33 \times C_{33}) / (C_{27} + C_{29} + C_{31} + C_{33})$

$ACL_{25\text{-}35}$ was calculated using the following formula:

$ACL_{25\text{-}35} = (25 \text{ x } C_{25} + 26 \text{ x } C_{26} + 27 \times C_{27} + 28 \text{ x } C_{28} + 29 \times C_{29} + 30\text{x } C_{30} + 31 \times C_{31} + 32 \text{ x } C_{32} + 33 \times C_{33} + 34 \text{ x } C_{34} + 35 \text{ x } C_{35}) / (C_{25} + C_{26} + C_{27} + C_{28} + C_{29} + C_{30} + C_{31} + C_{32} + C_{33} + C_{34} + C_{35})$

Several studies have suggested Norm31 and Norm33 as environmental parameters [e.g. 31, 78]. Norm31 is calculated as: Norm31=C_{31} / (C_{29} + C_{31}). Norm33 is calculated as: Norm33=C_{33} / (C_{29} + C_{33}). Norm33 related to water stress with higher water stress reflected in higher Norm33 values respectively, at least in a Mediterranean setting [79].

The odd-over-even predominance (OEP) was calculated using the following formula:

$OEP_{25\text{-}35} = (C_{25} + C_{27} + C_{29} + C_{31} + C_{33} + C_{35}) / (C_{26} + C_{28} + C_{30} + C_{32})$

after [43].

Table S1: Terminal Pleistocene and Holocene stratigraphic record from the 2018/2019 excavation seasons at Wonderwerk Cave [13] and proposed correlation to previously published layers [11, 53, 61].

2018/2019 Layers	Sediment description	Archaeological Stratum [11]
GS	Grey sand: Grey, dusty sandy layer with botanical remains, gravel and animal hair.	1a-c & 2a
FBS	Fine Brown Sand: tan coloured silty sand, includes bone fragments, caprine dung, banded ironstone formation clasts and little charcoal. Passage features indicate bioturbation.	2b & 3a
BWS	Brown With Speckles: Homogenous brown sand and silt with gypsum nodules. Bioturbation present. Ash and charcoal common,	3b
AS	Auburn Sand: Heterogenous fine sand and silt-sized quartz with many banded ironstone formation clasts.	4a
DL	Dark Lens: Heterogenous fine sand and silt-sized quartz with many banded ironstone formation clasts. No charcoal evident. Heavily insect bioturbated.	4aLH
AAS	Another Auburn Sand: Red-brown heterogenous fine sand and silt-sized quartz with many banded ironstone formation clasts, limestone, chert, seed coats and calcified roots. Small amount of charcoal. Green coloured clay casts. Bioturbation likely.	4b
CBS	Compacted Beige Sand: Beige-grey sand with patches of darker, looser sediment and prevalent banded ironstone formation inclusions.	4c
Not excavated		4d
Not excavated		5a/b

Table S2: Linear R pearson test (bottom/left) and *p* values (top/right) for all samples separated by biomarker indices and isotope values, including numbers of lithic artefacts and ostrich eggshell (OES) pieces per layer. The carbon isotope values for the modern sample and layer GS have been corrected by 2‰ to take a maximum correction regarding the Suess effect into account. Lithic and OES counts from [13]. Significant p-values (<0.05) are highlighted in green.

		ACL 25-35	CPI 25-35	OEP 25-35	lithic total	OES total	13C_nC_{27}	13C_nC_{29}	13C_nC_{31}	13C_nC_{33}	D_nC_{27}	D_nC_{29}	D_nC_{31}	D_nC_{33}
		A	B	C	D	E	F	G	H	I	J	K	M	N
ACL 25-35	A		0.137	0.160	0.192	0.082	0.446	0.015	0.037	0.920	0.072	0.204	0.570	0.034
CPI 25-35	B	-0.536		<0.000	0.119	0.003	0.956	0.363	0.246	0.048	0.047	0.064	0.473	0.716
OEP 25-35	C	-0.511	0.999		0.143	0.004	0.952	0.391	0.276	0.049	0.052	0.065	0.509	0.734
lithic total	D	-0.514	0.596	0.567		0.021	0.832	0.144	0.039	0.331	0.733	0.561	0.061	0.734
OES total	E	-0.648	0.896	0.882	0.783		0.883	0.421	0.296	0.120	0.157	0.391	0.229	0.847
13C_nC_{27}	F	-0.346	0.026	0.028	-0.112	0.078		0.563	0.870	0.497	0.706	0.804	0.303	0.710
13C_nC_{29}	G	-0.773	0.345	0.326	0.565	0.332	0.267		0.001	0.504	0.513	0.204	0.503	0.002
13C_nC_{31}	H	-0.698	0.431	0.408	0.733	0.423	-0.077	0.888		0.803	0.828	0.393	0.364	0.054
13C_nC_{33}	I	-0.039	0.671	0.670	0.396	0.594	-0.311	-0.257	0.097		0.834	0.904	0.387	0.361
D_nC_{27}	J	0.665	-0.713	-0.703	-0.159	-0.597	0.199	-0.273	-0.092	-0.089		0.009	0.356	0.492
D_nC_{29}	K	0.468	-0.639	-0.637	-0.244	-0.353	0.116	-0.468	-0.325	-0.047	0.839		0.946	0.422
D_nC_{31}	M	0.220	-0.275	-0.254	-0.685	-0.480	-0.457	-0.258	-0.344	-0.329	-0.378	-0.027		0.291
D_nC_{33}	N	-0.744	0.154	0.144	0.159	0.091	-0.196	0.905	0.699	-0.375	-0.315	-0.332	0.427	

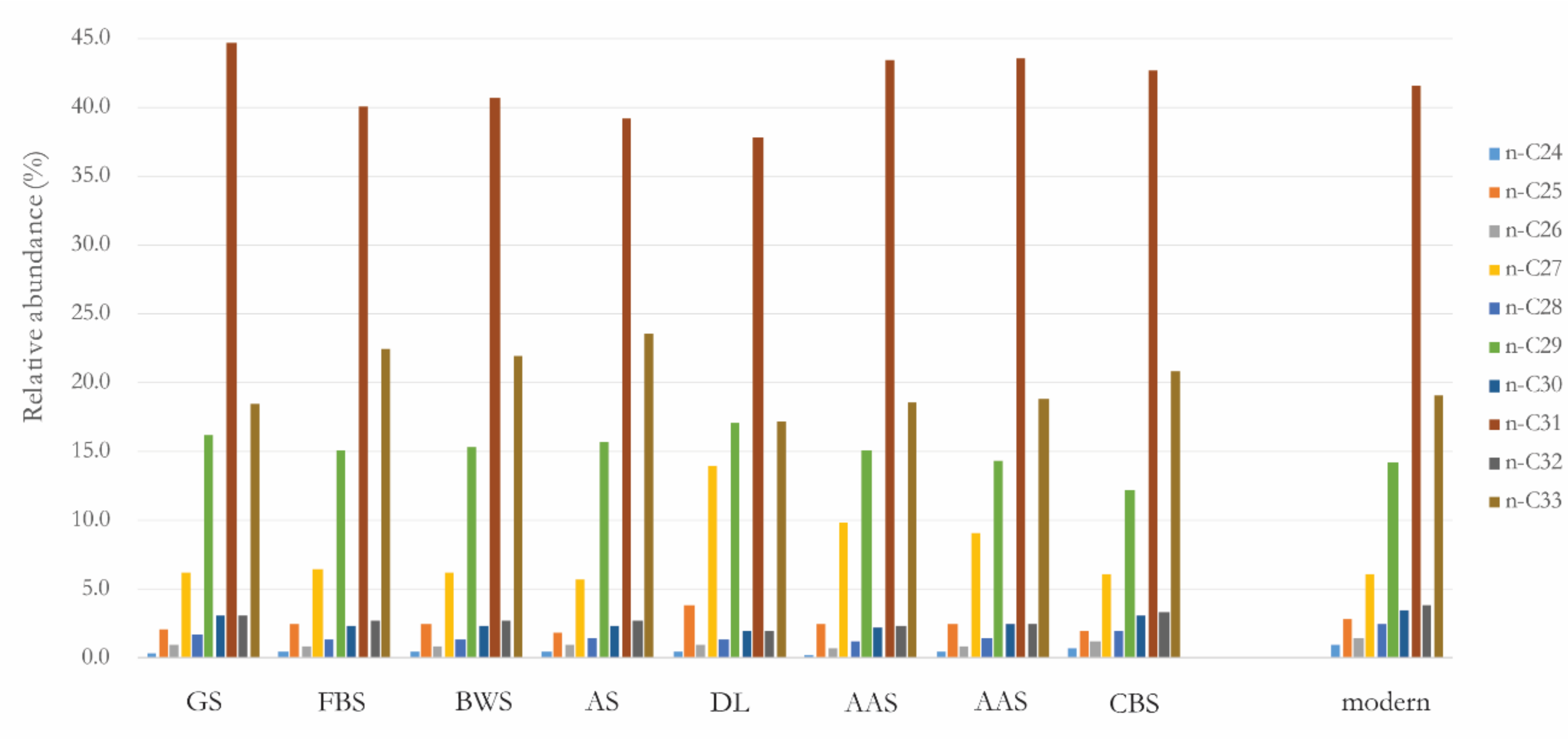

Figure S1. Relative abundance of *n*-alkane chains in the Wonderwerk Cave samples. Samples are ordered from youngest (GS) to oldest (CBS) sample, with the modern comparative sample set off on the righthand side.

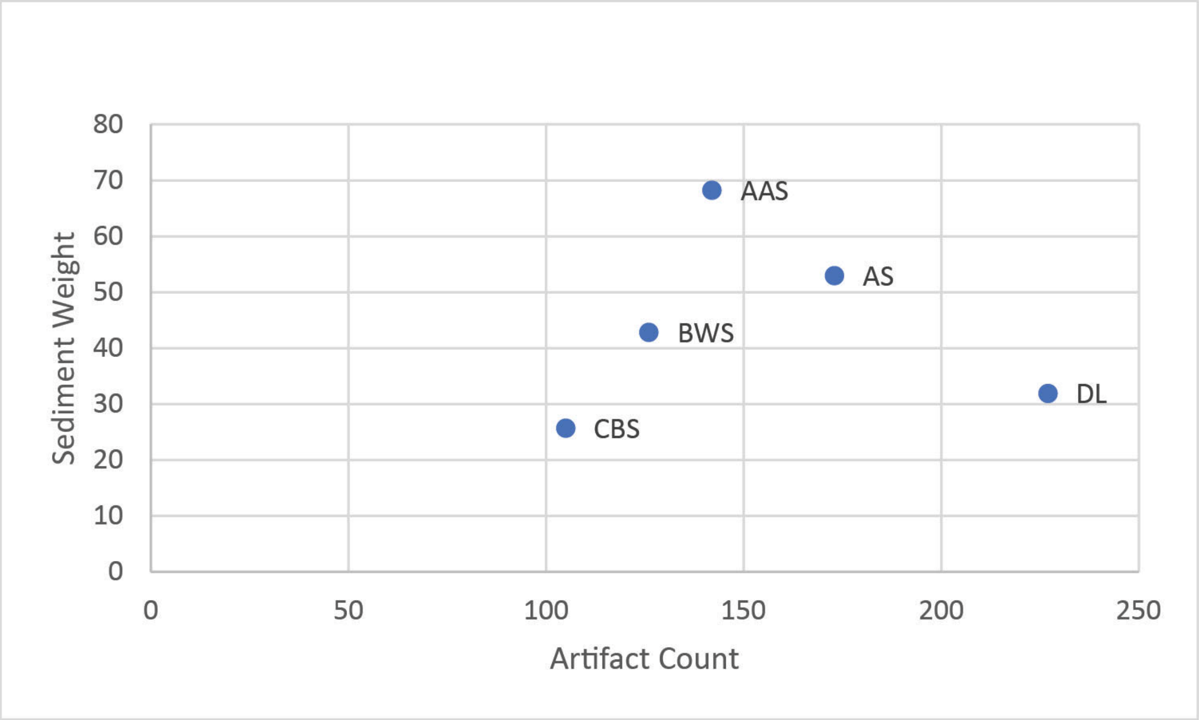

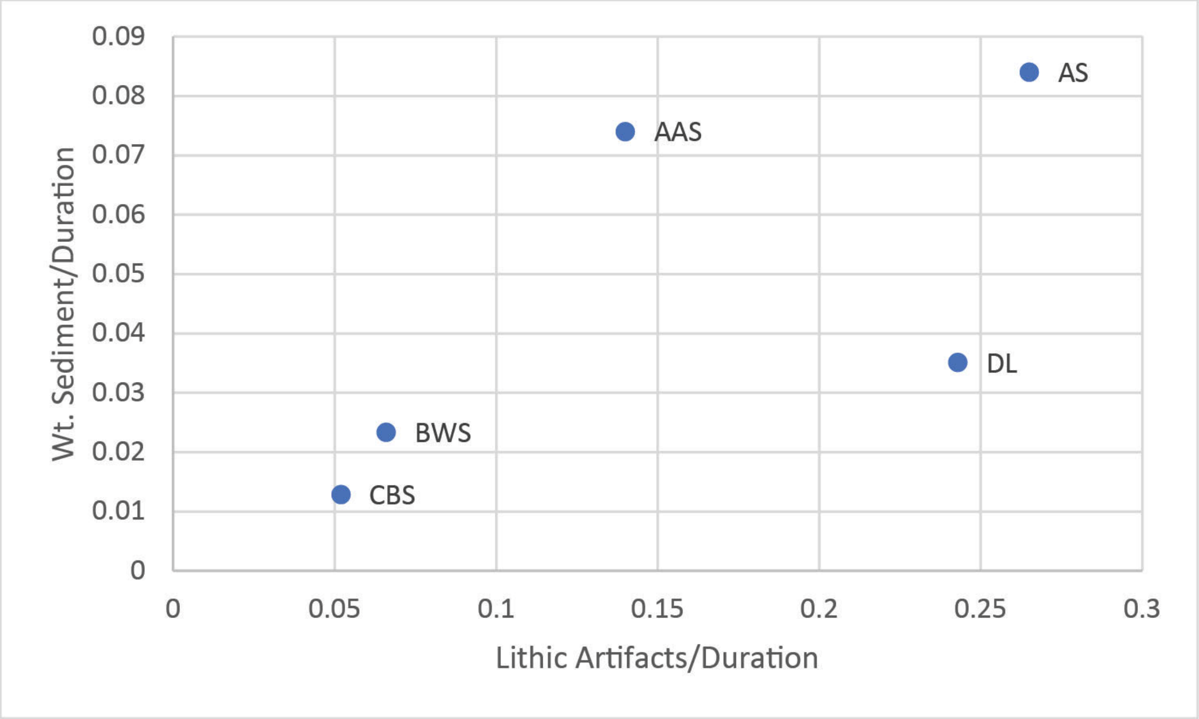

Figure S2: Above: Correlation of sediment weight in kg (based on weighed buckets during excavation), and number of lithic artefacts. Below: The same data time duration of each layer is taken into account. Duration is calculated based on the most likely start/end date for the layers based on the Bayesian model that assumes a series of sequential non-overlapping periods of deposition identified based on sediment characteristics (Table 1). In the case of Operation 5 there is no evidence

either from micromorphology or artefact distribution for significant hiatuses in human presence and sediment accumulation supporting the appropriateness of the use of this method to derive the temporal duration of each layer. No bucket weight data available for layer FBS.

Supplementary references

59. Malan, B. D. & Cooke, H. B. S. A Preliminary Account of the Wonderwerk Cave, Kuruman District. Section I: Site and Archaeology. *South African Journal of Science* **37,** 300–312 (1941).

60. Malan, B. D. & Wells, L. H. A Further Report on the Wonderwerk Cave, Kuruman. *South African Journal of Science* **40,** 258–270 (1943).

61. Thackeray, A. I. *The Holocene cultural sequence in the northern Cape Province, South Africa*. Unpublished dissertation, Yale University. (1981).

62. Bradfield, J., Thackeray, J.F. & Morris, D., An experimental investigation into the origin of incised lines on a 4ooo-year-old engraving from Wonderwerk cave, Northern Cape province. *South African Archaeological Bulletin* **69,** 72–79 (2014).

63. Chazan, M. Reframing the Wonderwerk slabs and the origins of art in Africa in *The Pasts and Presence of Art in South Africa. Technologies, Ontologies and Agents*, (eds. Wingfield, C., Giblin, J., King, R.) 21–30 (McDonald Institute for Archaeological Research, 2020).

64. Morris, D. Revisiting the Parietal Art of Wonderwerk Cave, South Africa. *African Archaeological Review* **33,** 265–275 (2016).

65. Hunter, A.A. & Gassner, B.R. Evaluation of the Flote-Tech machine-assisted flotation system. *American Antiquity* **63(1),** 143–156 (1998).

66. Klein, R.G., Cruz-Uribe, K. & Beaumont, P.B. Environmental, ecological, and paleoanthropological implications of the late Pleistocene mammalian fauna from Equus Cave, northern Cape Province, South Africa. *Quaternary research* **36,** 94–119 (1991).

67. Scott, L. Pollen analysis of hyena coprolites and sediments from Equus Cave, Taung, southern Kalahari (South Africa). *Quaternary research* **28,** 144–156 (1987).

68. Butzer, K.W., Stuckenrath, R., Bruzewicz, A.J. & Helgren, D.M. Late Cenozoic Paleoclimates of the Gaap Escarpment, Kalahari margin, South Africa. *Quat. res.* **10,** 310–339 (1978).

69. Lukich, V., Porat, N., Faershtein, G., Cowling, S. & Chazan, M., New Chronology and Stratigraphy for Kathu Pan 6, South Africa. *Journal of Paleolithic Archaeology* **2,** 235–257 (2019).

70. Eglinton, G., & Hamilton, R. J. Leaf Epicuticular Waxes: The waxy outer surfaces of most plants display a wide diversity of fine structure and chemical constituents. *Science* **156(3780),** 1322-1335 (1967).

71. Ehleringer, J. R. Carbon Isotope Ratios and Physiological Processes in Aridland Plants, In *Stable Isotopes in Ecological Research*, Vol. 68 Ecological Studies (eds Rundel, P. W., Ehleringer, J. R. & Nagy, K. A.) (1989).

72. O'Leary, M. Carbon isotope fractionation in plants. *Phytochemistry* **20,** 553–567 (1981).

73. Rommerskirchen, F., Plader, A., Eglinton, G., Chikaraishi, Y. & Rullkötter, J. Chemotaxonomic significance of distribution and stable carbon isotopic composition of long-chain alkanes and alkan-1-ols in C_4 grass waxes. *Org. Geochem*. **37,** 1303–1332 (2006).

74. Vogts, A., Moossen, H., Rommerskirchen, F. & Rullkötter, J. Distribution patterns and stable carbon isotopic composition of alkanes and alkan-1-ols from plant waxes of African rain forest and savanna C_3 species. *Org. Geochem*. **40,** 1037–1054 (2009).

75. Bi, X., Sheng, G., Liu, X., Li, C. & Fu, J. Molecular and carbon and hydrogen isotopic composition of *n*-alkanes in plant leaf waxes. *Organic Geochemistry* **36,** 1405–1417 (2005).

76. Sachse, D., Radke, J. & Gleixner, G. Hydrogen isotope ratios of recent lacustrine sedimentary *n*-alkanes record modern climate variability. *Geochimica et Cosmochimica Acta* **68(23),** 4877-4889 (2004).

77. Garcin, Y. et al. Hydrogen isotope ratios of lacustrine sedimentary *n*-alkanes as proxies of tropical African hydrology: insights from a calibration transect across Cameroon. *Geochimica et Cosmochimica Acta* ***79*****,** 106-126 (2012).

78. Smith, F. A. & Freeman, K. H. Influence of physiology and climate on δD of leaf wax *n*-alkanes from C_3 and C_4 grasses. *Geochim. Cosmochim. Acta* **70,** 1172–1187 (2006).

79. Schirrmacher, J., Weinelt, M., Blanz, T., Andersen, N., Salgueiro, E. & Schneider, R.R. Multi-decadal atmospheric and marine climate variability in southern Iberia during the mid- to late-Holocene. *Clim. Past* **15,** 617–634 (2019).
